# Endovascular treatment in Danon disease: a case report

**DOI:** 10.1186/s13256-024-04555-7

**Published:** 2024-05-12

**Authors:** Rayco Jiménez-Bolaños, Francisco Hernández-Fernández, Jorge García-García, Óscar Ayo-Martín, Laura del Rey Megias, Juan David Molina-Nuevo, Tomás Segura

**Affiliations:** 1 Department of Neurology, Complejo Hospitalario Universitario Insular-Materno Infantil de Canarias, Las Palmas, Spain; 2 Department of Neurology, Complejo Hospitalario Universitario de Albacete, C/Hermanos Falcó 37, 02006 Albacete, Spain; 3 Pediatric Cardiology, Department of Pediatrics, Complejo Hospitalario Universitario de Albacete, Albacete, Spain; 4 Department of Radiology, Complejo Hospitalario Universitario de Albacete, Albacete, Spain; 5 Institute for Research in Neurologic Disabilities (IDINE), Medical School, University of Castilla-La-Mancha, Albacete, Spain

**Keywords:** Danon, Stroke, Cardiomyopathy, Lysosomal, Case report

## Abstract

**Background:**

Danon disease is a lysosomal storage disorder with X-linked inheritance. The classic triad is severe hypertrophic cardiomyopathy, myopathy, and intellectual disability, with different phenotypes between both genders. Ischemic stroke is an uncommon complication, mostly cardioembolic, related to intraventricular thrombus or atrial fibrillation, among others.

**Case report:**

We report the case of a 14-year-old Caucasian male patient with Danon disease who suffered from an acute ischemic stroke due to occlusion in the M1 segment of the middle cerebral artery. He underwent mechanical thrombectomy, resulting in successful revascularization with satisfactory clinical outcome. We objectified the intraventricular thrombus in the absence of arrhythmic events.

**Conclusion:**

To our knowledge, we report the first case of ischemic stroke related to Danon disease treated with endovascular treatment.

## Background

Danon disease (DD) is a lysosomal storage disorder caused by the mutation of the Lysosomal Associated Membrane Protein 2 (*LAMP-2*) gene. The exact LAMP-2 protein functions are unknown; however, it seems to play an important role in autophagy [1]. Its inheritance is X-linked dominant, located in Xq24, with more than 20 mutations described. Severe cardiomyopathy and myopathy are typical features, commonly associated with mental disability. Nevertheless, isolated cardiomyopathy is not infrequent [2]. Generally, men develop symptoms earlier and more intensely than women [3]. The prevalence of DD is unknown, but it is estimated at 0.7% of adult patients with hypertrophic cardiomyopathy (HCM) [7]. To date, approximately 500 cases have been described [3].

Early morbidity and mortality due to heart failure are known to occur in DD. Furthermore, cardioembolic complications may be relatively common in these patients even with normal systolic function. Indeed, one of the more devastating complications in the natural evolution of patients with DD is the onset of an ischemic stroke secondary to atrial fibrillation (AF) [4] or intracardiac thrombi, located in the left ventricle [3, 5]. Formation of these thrombi is uncommon in non-metabolic HCM except when associated with severe ventricular dysfunction or AF or Wolff-Parkinson-White (WPW) arrhythmias, which may often occur in patients with DD. There is a lack of research on the management of acute stroke in these patients.

We present the first case of a patient with DD who suffered an acute ischemic stroke and treated with mechanical thrombectomy.

## Case report

### Medical history

An 18-month-old Caucasian boy, with no relevant prenatal, medical, or family history, parents not consanguineous, suffered from delayed motor development, hypotonia, and an increase in creatine-kinase and liver enzymes. The patient developed progressive proximal muscle weakness in both upper and lower limbs with bilateral winged scapula. Electromyography revealed a myopathic pattern. During follow-up at 10 years, after identifying a heart murmur, echocardiography revealed severe global left ventricular hypertrophy, mainly at the intraventricular septum (IVS 14–15 mm), with multiple trabeculae and preserved systolic function. The electrocardiogram showed WPW syndrome. The muscle biopsy reported vacuolar myopathy with autophagic characteristics and glycogen accumulation. The genetic test confirmed the presence of a mutation c.973dup;p (leu325profs25) in the LAMP-2 gene. An implantable cardioverter defibrillator was inserted due to atrioventricular block.

## Case history

At the age of 14 years, he experienced speech impairment and right limb weakness upon awakening. Physical examination showed aortic systolic murmur and normal vital signs (blood pressure 116/81 mmHg; heart rate 60 beats per minute; temperature 36 °C). Neurological examination revealed moderate dysarthria, right central facial paresis and ipsilateral hemiparesis, right Babinski reflex and left brachial hypesthesia. The National Institute of Health Stroke Scale (NIHSS) score was 9.

## Diagnostic investigations and endovascular procedure

**Fig. 1:**
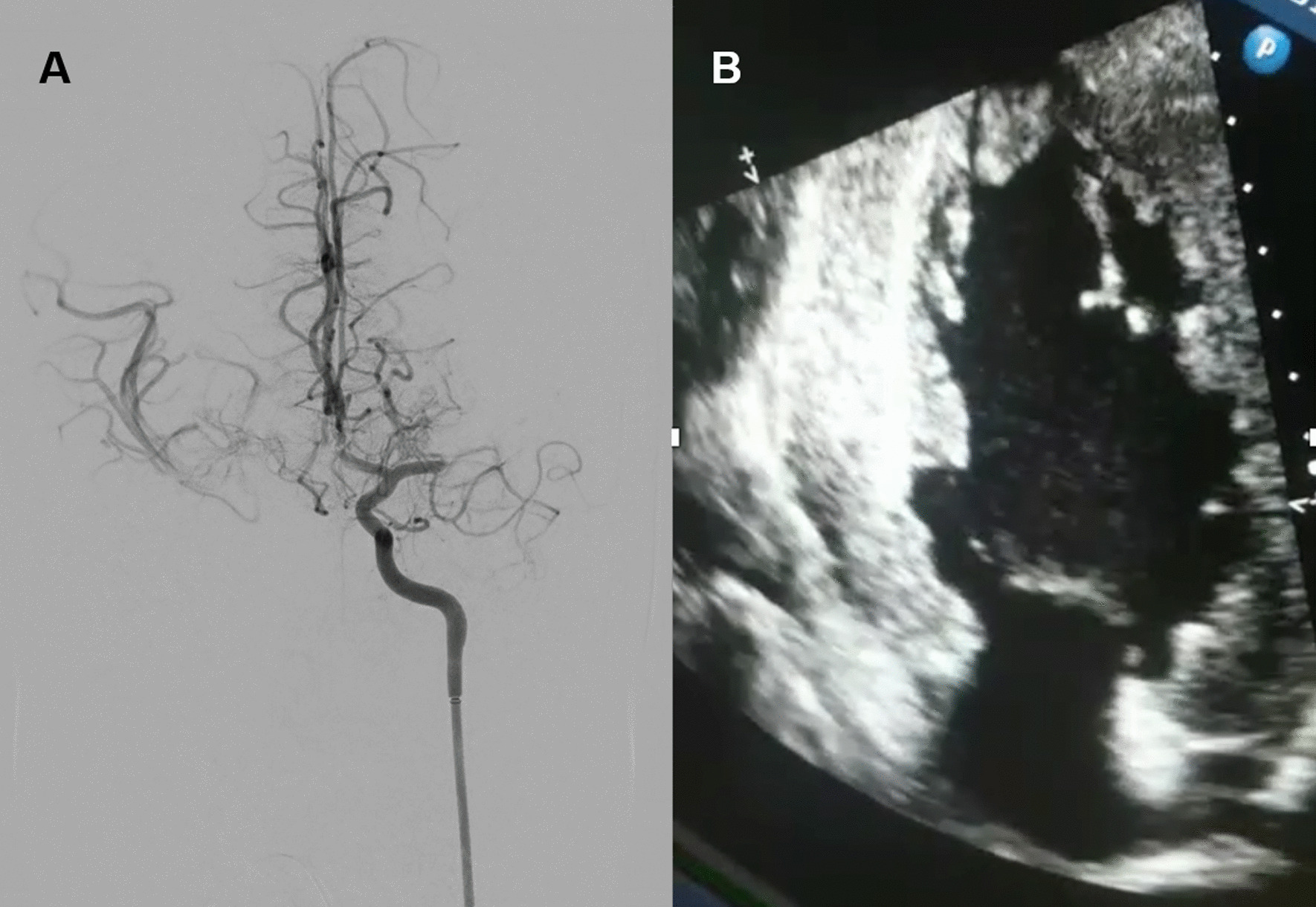
**A** Cerebral angiography during procedure demonstrating a complete occlusion of the left middle cerebral artery bifurcation. **B** Echocardiogram revealed a hypertrophic cardiomyopathy and an echogenic mass at the left ventricular apex suggestive of intracardiac thrombus

A head computed tomography (CT) scan and CT angiogram demonstrated an Alberta Stroke Program Early CT Score (ASPECTS) of 10 and a complete occlusion of the left middle cerebral artery (MCA) bifurcation. A CT-perfusion study showed a large area of long mean transit time (MTT) with complete conservation of cerebral blood volume (CBV) in the left frontal-parietal-temporal territory. According to the local ischemic stroke protocol, the patient met the criteria for mechanical thrombectomy, except for age (less than 18 years) and DD. Therefore, due to excellent prognostic factors (ASPECTS 10 and favorable mismatch), emergent primary endovascular treatment was decided upon after informing the parents. Mechanical thrombectomy was performed using stent retriever with complete flow restoration (TICI 3) in one pass (Fig. 1A).

## Post-procedure care and etiological evaluation

The patient was admitted to the stroke unit and became asymptomatic 24 hours post-procedure (NIHSS 0). The CT scan evidenced hypo-attenuation of left caudate and lentiform nuclei and anterior limb of internal capsule. An etiological assessment was performed and an echocardiogram revealed worsening of the left ventricular hypertrophy (IVS 30–35 mm) showing an echogenic mass at the left ventricular apex suggestive of intracardiac thrombus (Fig. 1B), secondary to heart disease previously observed during follow-up. The laboratory findings showed an increased in creatine-kinase (CK 1596 μmol/l) and liver enzymes glutamate pyruvate alanine aminotransferase (GPT 274 U/L), similar to previous values, without other alterations (glucose 106 mg/dL; urea 25 mg/dL; creatinine 0.62 mg/dL; sodium 141 mEq/L; potassium 4 mEq/L; chlorine 103 mEq/L). Urinary sediment analysis and toxin screen were negative. The coagulation test was normal, including international normalized ratio (INR) 1.36. We initiated low-molecular weight heparin (enoxaparin 60 mg/12 hours) for 15 days as bridging therapy despite the determination of a factor VII deficiency. Subsequently, we replaced apixaban 5 mg/12 hours instead of vitamin-K antagonist due to high bleeding risk. On discharge, the patient had completely recovered to his prior neurological state, which included winged scapulae and waddling gait. The patient remained asymptomatic during the 6-month follow-up and the apical thrombus disappeared.

## Discussion and conclusions

DD may be a rare cause of ischemic stroke due to a cardioembolic mechanism, even in those with normal systolic function. In selected cases based on the main criteria, endovascular treatment may be an effective therapeutic strategy to prevent disability in patients with large-vessel-occlusion ischemic stroke, but further research is needed to improve upon knowledge.

DD is a rare multisystem disorder characterized by the triad of HCM, skeletal myopathy and intellectual disability. Cardiac involvement typically described is HCM; nevertheless, dilated cardiomyopathy has also been reported, mainly in women [2]. The X-linked dominant inheritance involves differences in clinical severity between both genders. Women usually develop a milder clinical phenotype, and occasionally only have heart disease, which can be as serious as in men [3]. Women usually develop a milder clinical phenotype, though with an exclusive cardiac involvement with similar damage.

DD prevalence is unknown. Around 500 cases have been reported worldwide since it was first described first in 1981 [3]. Many patients develop ischemic stroke, frequently at younger ages and with unfavorable prognosis. No endovascular treatment in acute event has been informed [3].

In 2008, Spinazzi *et al*. described three patients (two men and one woman) with severe HCM and AF who suffered from ischemic stroke [4]. In 2016, Marino *et al*. described a patient with hemodynamic ischemic stroke following cardiac arrest secondary to WPW [6]. In 2017, Takeshi *et al*. described a patient with ischemic stroke secondary to intracardiac thrombus [5]. Embolic stroke from the heart is common in DD and is associated with left ventricular dysfunction, older age, AF, or congestive heart failure [4]. Intracardiac thrombus formation is very rare in HCM, unless associated with very severe left ventricular dysfunction, AF, or WPW syndrome. Early identification of embolic source is relevant for prompt initiation of anticoagulation if indicated [3, 5]. In our case, we established the cardioembolic source of the ischemic stroke after demonstrating an intraventricular thrombus on echocardiography.

To our knowledge, we present the first case of an acute ischemic stroke in a patient with DD who received mechanical thrombectomy. We performed the most widely used extraction method, that is, the stent retriever, with excellent angiographic and clinical results.

Therefore, it is reasonable to consider endovascular treatment beneficial in these patients. In addition, it is the most efficient therapeutic tool to prevent disability secondary to ischemic stroke. The development of impairment may contraindicate cardiac transplantation, therefore avoiding disability should be of major importance. Nonetheless, further studies are necessary to confirm our results.

## Data Availability

The datasets used and/or analyzed during the current study are available from the corresponding author on reasonable request.

## References

[1] Danon M, Oh S, DiMauro S, Manaligod J, Eastwood A, Naidu S (1981). Lysosomal glycogen storage disease with normal acid maltase. Neurology.

[2] López-Sainz A, Salazar-Mendiguchía J, García-Álvarez A, Campuzano Larrea O, López-Garrido MA, García-Guereta L (2019). Clinical findings and prognosis of Danon disease an analysis of the Spanish Multicenter Danon Registry. Rev Esp Cardiol..

[3] Cenacchi G, Papa V, Pegoraro V, Marozzo R, Fanin M, Angelini C (2019). Review: Danon disease: review of natural history and recent advances. Neuropathol Appl Neurobiol.

[4] Spinazzi M, Fanin M, Melacini P, Nascimbeni A, Angelini C (2008). Cardioembolic stroke in Danon disease. Clin Genet.

[5] Tsuda T, Shillingford A, Vetter J, Kandula V, Jain B, Temple J (2017). Transient ischemic attack and ischemic stroke in Danon disease with formation of left ventricular apical thrombus despite normal systolic function. Case Rep Pediatr.

[6] Marino M, Musumeci O, Paleologo G, Cucinotta M, Migliorato A, Rodolico C (2016). Ischemic stroke due to hypoperfusion in a patient with a previously unrecognized Danon disease. Neuromuscul Disord.

[7] Mazzarotto F, Girolami F, Boschi B, Barlocco F, Tomberli A, Baldini K (2019). Defining the diagnostic effectiveness of genes for inclusion in panels: the experience of two decades of genetic testing for hypertrophic cardiomyopathy at a single center. Genet Med.

